# Nanomedicine: A Diagnostic and Therapeutic Approach to COVID-19

**DOI:** 10.3389/fmed.2021.648005

**Published:** 2021-06-04

**Authors:** Arjun Sharma, Konstantinos Kontodimas, Markus Bosmann

**Affiliations:** ^1^Pulmonary Center, Department of Medicine, Boston University School of Medicine, Boston, MA, United States; ^2^Center for Thrombosis and Hemostasis, University Medical Center of the Johannes Gutenberg-University, Mainz, Germany

**Keywords:** SARS-CoV-2 virus, vaccine, nanotechnology, drug delivery systems, sepsis, acute respiratory distress syndrome

## Abstract

The SARS-CoV-2 virus is causing devastating morbidity and mortality worldwide. Nanomedicine approaches have a high potential to enhance conventional diagnostics, drugs and vaccines. In fact, lipid nanoparticle/mRNA vaccines are already widely used to protect from COVID-19. In this review, we present an overview of the taxonomy, structure, variants of concern, epidemiology, pathophysiology and detection methods of SARS-CoV-2. The efforts of repurposing, tailoring, and adapting pre-existing medications to battle COVID-19 and the state of vaccine developments are presented. Next, we discuss the broad concepts and limitations of how nanomedicine could address the COVID-19 threat. Nanomaterials are particles in the nanometer scale (10–100 nm) which possess unique properties related to their size, polarity, structural and chemical composition. Nanoparticles can be composed of precious metals (copper, silver, gold), inorganic materials (graphene, silicon), proteins, carbohydrates, lipids, RNA/DNA, or conjugates, combinations and polymers of all of the aforementioned. The advanced biochemical features of these nanoscale particles allow them to directly interact with virions and irreversibly disrupt their structure, which can render a virus incapable of replicating within the host. Virus-neutralizing coats and surfaces impregnated with nanomaterials can enhance personal protective equipment, hand sanitizers and air filter systems. Nanoparticles can enhance drug-based therapies by optimizing uptake, stability, target cell-specific delivery, and magnetic properties. In fact, recent studies have highlighted the potential of nanoparticles in different aspects of the fight against SARS-CoV-2, such as enhancing biosensors and diagnostic tests, drug therapies, designing new delivery mechanisms, and optimizing vaccines. This article summarizes the ongoing research on diagnostic strategies, treatments, and vaccines for COVID-19, while emphasizing the potential of nanoparticle-based pharmaceuticals and vaccines.

## Introduction

Severe Acute Respiratory Syndrome Coronavirus 2 (SARS-CoV-2) infection causes the ongoing pandemic of Coronavirus Disease 2019 (COVID-19). In 2019, the first confirmed and documented cases of COVID-19 in China rapidly progressed to a worldwide state of emergency unparalleled since the outbreak of the Spanish Flu in 1918. The failure to control the spread of COVID-19 has highlighted the urgency of developing diagnostic and therapeutic approaches against highly contagious pathogens. A plethora of innovative treatments is being proposed which incorporate the use of traditional and futuristic methods to minimize the pathogenicity, morbidity and mortality of SARS-CoV-2. Nanotechnology is an emerging field that has branched into the world of medicine. Due to its progressive nature, nanomedicine can overcome difficulties facing conventional medicine. Most importantly, it will hopefully contribute to revolutionizing drug-based medicine in the twenty first century.

Nanomaterials have properties that, if exploited correctly, may improve treatments and vaccines, and provide alternative and safer ways to battle diseases (1). However, emergence of side effects of these nanoparticles, such as unwanted interactions with tissues or increased inflammation, could put a temporary hold on the utilization of nanotechnology (2). The COVID-19 crisis sets the stage to evolve the concepts of nanotechnology into reality. As its potential is revealed, it can offer innovative ways of protecting healthy and infected individuals, detecting SARS-CoV-2, and helping to end the pandemic.

In this review, we present an overview of SARS-CoV-2 pathophysiology, diagnostics, treatment and vaccines followed by discussing the current and future applications of nanomedicine aiming to mitigate the COVID-19 pandemic. The nanoparticle approaches presented here will help to win the fight against SARS-CoV-2 and other pathogens.

## SARS-COV-2

### Origin and Transmission

**Figure 1:**
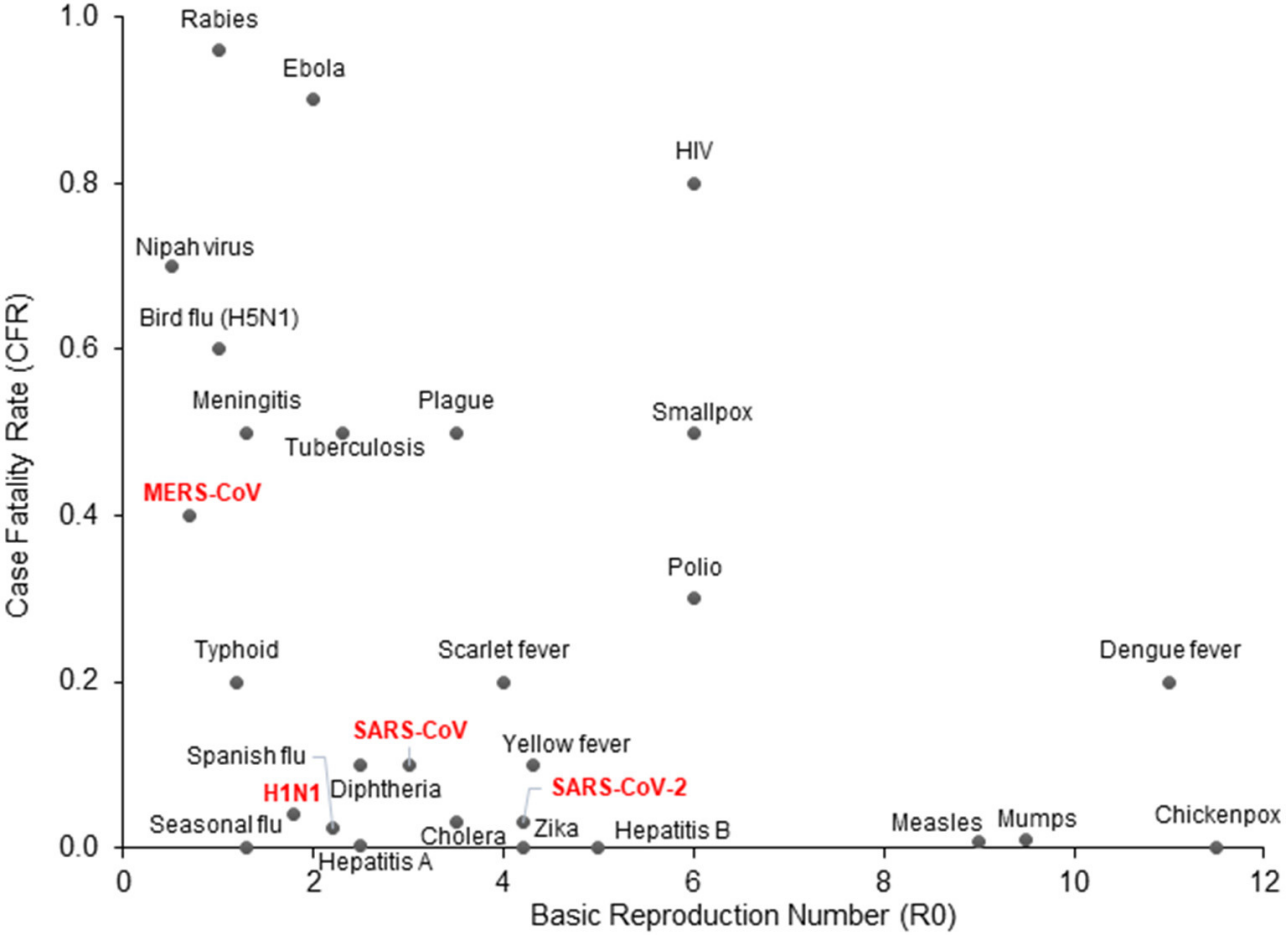
COVID-19 epidemiologic characteristics compared to other prevalent infections. The Case Fatality Rate (CFR) for COVID-19 is estimated around 2–4% with some variation and a recent decline due to optimized supportive care. The Basic Reproduction Number (R0) shown on the x-axis is also an estimate from epidemiological data. SARS-CoV-2 is more contagious than SARS-CoV and MERS-CoV, which may be attributed to longer incubation periods and asymptomatic carriers.

In the first week of January 2020, the Chinese Center for Disease Control and Prevention (CCDC) disclosed that 27 cases of pneumonia admitted during late December of 2019, were attributed to Severe Acute Respiratory Syndrome Coronavirus 2 (SARS-CoV-2), later named COVID-19 by the World Health Organization (WHO) (3). The patients had visited one of the “wet markets” in Wuhan city, located in China's Hubei province, which are known for their considerable variety of wild animals for sale (4). Recent genomic analysis has revealed that the SARS-CoV-2 genome is 96% identical to a known bat coronavirus (BatCoV RaTG13) from *Rhinolophus affinis*, a species found in Yunnan province (5, 6). The WHO declared the viral outbreak a public health emergency of global proportions at the end of January, when there were approximately 10,000 diagnosed cases around the globe (7). It was estimated that SARS-CoV-2 has a Case Fatality Rate (CFR) of 2–4% (8, 9) with substantial variation between countries, as well as a higher basic reproduction number (median R_0_ range: 3.5–4.7) compared to other coronaviruses or influenza (Figure 1) (10–12). As of April 2021, more than 140 million people across the globe have contracted COVID-19, and more than 3 million of those cases resulted in fatalities.

### Taxonomy and Structure

Coronaviruses (CoVs) belong to the *Cornidovirineae* suborder under the *Coronaviridae* family. CoVs are a predominant group of viruses, but of the 46 known CoVs only 7 have been confirmed to infect humans (13). Human coronavirus 229E (HCoV-229E) and human coronavirus NL63 (HCoV-NL63) are members of the genus *Alphacoronavirus* while human coronavirus OC43 (HCoV-OC43) and human coronavirus HKU1 (HCoV-HKU1) belong to the genus *Betacoronavirus*. These viruses are linked to mild upper respiratory tract diseases and can be attributed to 15–30% of common cold cases with regional/global and seasonal patterns (Table 1) (14, 22, 23). In contrast, SARS-CoV (sometimes referred to as SARS-CoV-1), MERS-CoV, and SARS-CoV-2 of the *Betacoronavirus* genus, are associated with severe disease pathophysiology, including respiratory disease, multi-organ failure, sepsis and death (14–16, 20, 21, 24–26).

**Table 1:** Human pathogenic coronaviruses.

**Name**	**R0**	**CFR**	**Pathophysiology**	**Natural host–intermediate host**	**Epidemiology**	**References**
HCoV-229E	NA	NA	Sore throat, Fever, Cough, Headache, Nasal discharge	Bats–Camelids?	Global–Fall	(13)
HCoV-NL63	NA	NA	Cough, Fever, Hypoxia, Tachypnea	Bats–NA	Global–Fall	(14)
HCoV-OC43	NA	NA	Sore throat, Fever, Cough, Headache, Nasal discharge	Rodents–Bovines	Global–Fall	(15)
HCoV-HKU1	NA	NA	Fever, Cough	Rodents–NA	Global–Fall	(16)
MERS-CoV	0.7	0.4	Pneumonia, Sore throat, Fever, Cough, Chills, Dyspnea	Bats–Camels	Middle East−2011	(12, 17)
SARS-CoV	3	0.1	Respiratory distress, Fever, Dry cough, Headache, Myalgia	Bats–Palm Civets	China then Global−2003	(12, 18)
SARS-CoV-2	3.5–4.7	0.03	Pneumonia, ARDS, Fibrosis, Fever, Dry cough, Coagulopathy	Bats–Pangolins?	China then Global−2019	(7, 10, 19)
H1N1	1.7	0.04	Cough, Sore throat, Chills, Fever, Headache	Pigs–Pigs	Global–Fall (Outbreak 2011)	(12, 20, 21)

*SARS-CoV, MERS-CoV, and SARS-CoV-2 can cause severe morbidity and mortality in vulnerable individuals. Infections with other coronaviruses usually only result in mild symptoms. For comparison, the influenza A subtype, H1N1, of the orthomyxovirus family is shown. R0, Basic reproduction number; CFR, Case fatality rate; NA, Not available*.

**Figure 2:**
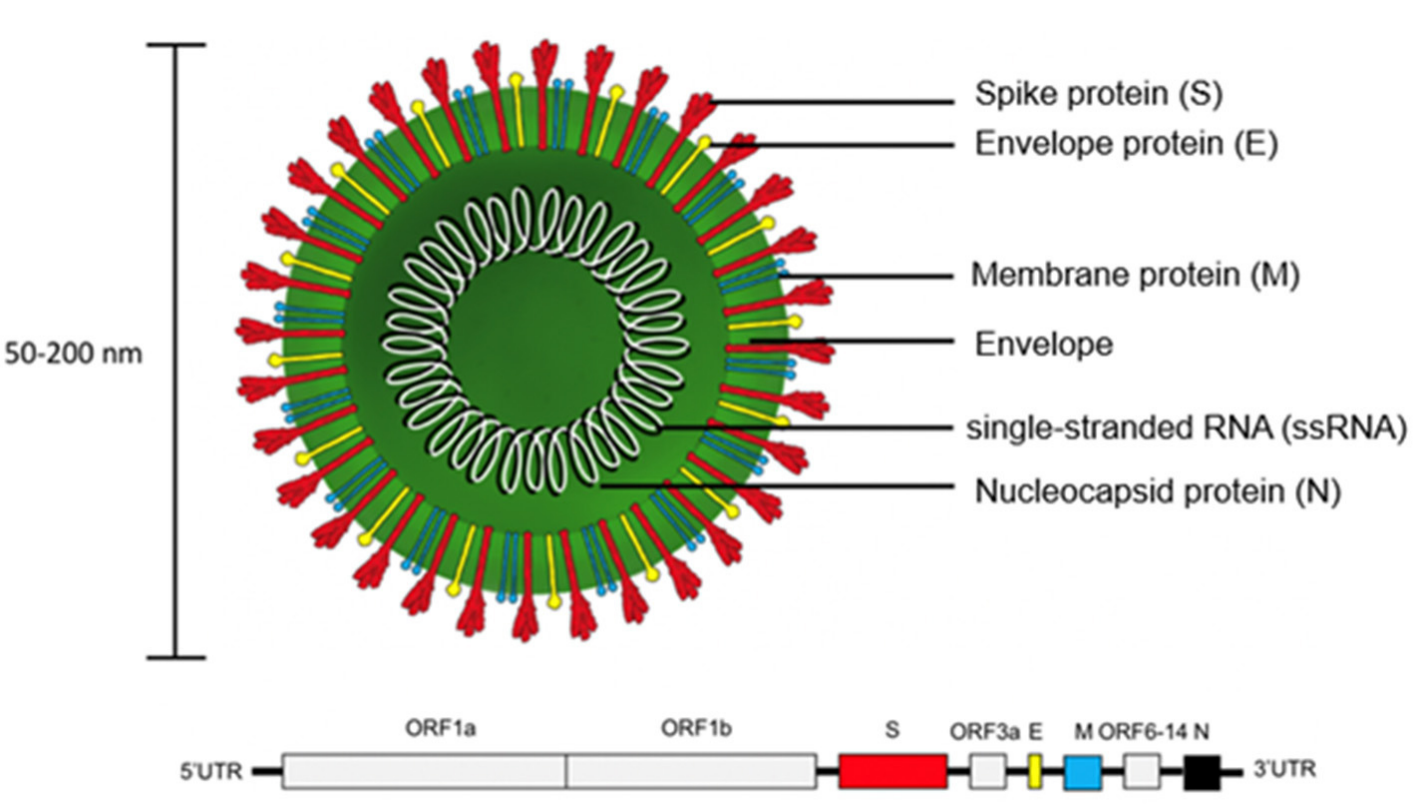
Structure of SARS-CoV-2 virion and genome. The trimeric spike protein (S) is required for docking to the hACE2 receptor. S protein is targeted by antibody-based therapies and is used as the immunogen for vaccine candidates. The single-stranded RNA (ssRNA) genome is bound to the Nucleocapsid phosphoprotein (N) which facilitates transcription after virus entry into host cells. The large viral genome (29.9 kb) is arranged as open reading frames (ORF) encoding for about 27 non-structural proteins (e.g., replicase, protease) and the four structural proteins (S, E, M, N).

SARS-CoV-2 is encoded by positive single-stranded RNA (ssRNA) bound to the nucleocapsid phosphoprotein (N). It is enclosed in a bilipid envelope surrounded by transmembrane proteins, such as the small envelope glycoprotein (E), membrane glycoprotein (M), and type-I trimeric spike protein (S) (Figure 2) (27–33). SARS-CoV-2 spike protein binds the Angiotensin Converting Enzyme 2 (ACE2) receptor located on type I and II pneumocytes and other epithelial and non-epithelial tissues, to enter host cells (34). More specifically, the spike protein monomers depend on host proteases for entry, such as the transmembrane serine protease 2 (TMPRSS2). TMPRSS2 can hydrolyze peptide bonds between the S1 and S2 subunits (35, 36). This process primes the spike protein and allows the S1 subunit, which contains the receptor binding domain (RBD) held together by several disulfide bonds, to bind with the N-terminal helix of ACE2 (17, 18, 37, 38). After internalization into the host cell, SARS-CoV-2 undergoes an uncoating process and initial viral transcription which requires supportive proteins and enzymes, including some rarely found in other RNA viruses such as (3'-to-5' exoribonuclease, 2'-O-ribose methyltransferase, ADP ribose 1'-phosphatase) (27). The viral transcripts can amass to 15–30% of the transcriptome in infected host cells (39). The translation of viral proteins occurs in the cytoplasm and viral proteins control the replication process. Viral proteins are inserted into the Golgi apparatus and are transported to the plasma membrane, where virions are released and begin infecting neighboring cells (19).

The genome sequences by next-generation sequencing (NGS) indicated that SARS-CoV-2 is more closely related to bat coronaviruses (BatCoV RaTG13 [96%], SL-CoVZXC21 [88%], SL-CoVZC45 [88%] (5), than to SARS-CoV (79% similarity) and MERS-CoV (50% similarity) (27, 40). SARS-CoV-2 spike protein has ~75% sequence similarity to the amino acid sequence of SARS-CoV spike protein (41), including a mutation in the C-terminal RBD for enhanced binding to ACE2 (5, 42, 43). SARS-CoV-2 quasispecies have been described, although the mutation rate is slower than for influenza virus (44). Virus variants of concern with higher infectivity and pathogenicity and a risk for resistance against the first generation of vaccines have emerged (45). A variant encoding a D614G mutation (conversion of aspartic acid to glycine at position 614) in the spike protein, located in the S1 domain has become most prevalent (46). This new D614G variant is associated with increased replication and transmission when compared to other less common isolates, such as the USA-WA1/2020 variant, which contains an aspartic acid residue at this position (47, 48). There are several sub-variants, such as the D16 INMI1 isolated in Italy, the G614 PV08449/2020 isolated in New York and the G614 BavPat1/2020 isolated in Germany (49, 50). Three variants of concern each with 17 amino acid changes and all featuring a N501Y spike protein mutation have emerged in the end of 2020 (51): A VUI-202012/01 (B.1.1.7) variant was first detected in the United Kingdom (52). The 501Y.V2 (B.1.351) variant was first discovered in South Africa and the P.1 variant was initially reported in Brazil and Japan (53). The P.1 and B.1.351 variants contain an E484K spike mutation.

### Pathophysiology

The clinical presentation of COVID-19 can be grouped in three categories based on disease severity and progression: the asymptomatic phase/stage, the mild symptomatic stage, and the severe respiratory infection stage (Table 2). Most individuals do not pass through all stages and asymptomatic or mild symptoms are most common (61). It is estimated that 15–30% of cases are asymptomatic, which may contribute to herd immunity (62). Individuals in the first category, also known as “stealth carriers,” do not present any symptoms and molecular testing can even be negative. If COVID-19 progresses to stage 2, mild infection symptoms are observed such as fever and coughing, and the patient typically tests positive in RT-PCR assays (59). It can take 1–3 weeks after the first symptoms for the production of antibodies against SARS-CoV-2. The third and most severe phase may present as a flu-like stage, a respiratory inflammation stage including pneumonia, acute respiratory distress syndrome (ARDS), pulmonary edema, and sometimes the complications of coagulopathy and fibrotic changes due to lung remodeling (54). This sequence of events can result in dramatically compromised gas exchange and respiratory failure (Table 2) (55, 63). Severe COVID-19 (stage 3) appears to be associated with a higher production of neutralizing antibodies. Additional symptoms include gastrointestinal dysfunction and secondary infections, as well as harmful tissue destruction due to pro-inflammatory leukocytes such as macrophages and granulocytes (56, 58–60).

**Table 2:** COVID-19 pathophysiology.

	**Observed symptoms**	**Clinical markers**	**Viral burden**	**Immune response**	**Therapeutic strategy**
Stage 1 Early infection phase	None or mild symptoms	Lymphopenia, ↑CRP and ↑IL-6	Low (Incubation period)	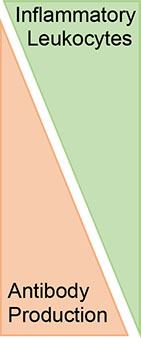	No therapy needed
Stage 2 Pulmonary phase	Dry coughing, Fever, Shortness of breath, Headache	Glass opacities (CT scans), Mild hypoxia	Intermediate (Spread from lower respiratory tract)		Treat symptoms
Stage 3 Hyperinflammation phase	Pneumonia, Chest pain, Productive coughing, Multiple organ failure	Cytokine storm, ARDS, Severe hypoxemia, Acute kidney injury	Highest (Expansion throughout the respiratory tract)		Reduce inflammation, Mechanical ventilation, Hemodialysis
References	(54–57)	(54, 55, 58)	(59)	(54, 60)	(55, 60)

*The time course and severity of illness can be classified into three stages (1–3). The clinical presentations are variable and most patients do not experience all stages*.

Emerging evidence suggests that a previous infection with one of the four endemic coronaviruses that cause “common cold” (HCoV-OC43, -HKU1, -NL63, and−229E) is associated with mitigated SARS-CoV-2 illness, which may be explained by a better pre-existing immune response and heterotypic immunity to homologous viruses (64). In addition to neutralizing, cross-reactive antibodies, memory CD4^+^ T cells have been hypothesized to reduce lung viral burden, accelerate antibody production and to enable heterotypic immunity (65–67). On the other hand, anti-SARS-CoV-2 antibodies with cross-reactivity for host proteins may contribute to pathologies such as Kawasaki-like disease and Guillain-Barré syndrome (68–70).

Many factors can influence the severity and outcome of COVID-19 infection such as age, gender, pre-existing health conditions and comorbidities (71). In general, the rates for apparent infection, hospitalization and death are higher for individuals aged 65 and above. Men have a higher risk for severe disease, an observation that has not been fully explained (72, 73). One hypothesis is centered around the higher tobacco use in men (4:1) and that long-term smokers develop cardiovascular and respiratory conditions which correlate with rapid and severe progression of COVID-19 (57). Increased vulnerability to SARS-CoV-2 is also correlated with a variety of health factors such as severe obesity, type II diabetes mellitus, serious cardiovascular conditions and immunocompromised states such as autoimmune disease or recent chemotherapy (74, 75). Last but not least, susceptibility to COVID-19 has been linked to certain genetic traits including polymorphisms for *IFNAR2, TYK*2*, TLR7, OAS1, DPP9*, and *CCR2*, and the major histocompatibility complex loci (*HLA*) (76) which also provide susceptibility to other infections such as Influenza, Hepatitis B, and leprosy (77).

### Research and Detection

The WHO and the Center for Disease Control and Prevention (CDC) have established detailed protocols regarding the use, containment, culturing, and testing for SARS-CoV-2. The CDC has classified any research work with infective SARS-CoV-2 as Biosafety Level 3 (BSL-3) category, while protocols with inactivated forms of SARS-CoV-2 or pseudotyped viruses can be performed in a BSL-2 laboratory (78).

It is widely accepted that suitable cell lines to propagate SARS-CoV-2 must express sufficient numbers of ACE2 and TMPRSS2 on their surface. The Vero cell line is derived from kidney cells of the African green monkey and sublineages such as the Vero E6 and Vero CCL81 cell lines produce even higher SARS-CoV-2 titers. Other cell types such Calu-3 (a human lung cancer line), Caco-2 (a human colorectal adenocarcinoma line), HEK 293T (derived human embryonic kidney line) and Huh7 (a human hepatocellular carcinoma line) can also be used for infection studies, but are not suitable for generating high titer virus stocks (79). Genetically modified cell lines, such as an ACE2 overexpressing HEK 293T line and Air-Liquid Interface (ALI) epithelial cell models exist (80). Human induced Pluripotent Stem Cell (iPSC)-derived alveolar type 2 cells (iAT2) are susceptible to SARS-CoV-2 infection in ALI culture. SARS-CoV-2 infection of the iAT2 cells hijacks the transcriptomic machinery, deprograms host cell differentiation, while inducing the NFkB pathway and interferon-dependent host defense programs (39, 81).

SARS-CoV-2 infection can be investigated in animal models. Mice *(Mus musculus)* were genetically engineered to over-express human ACE2 because SARS-CoV-2 spike protein does not bind well to murine ACE2. In addition, non-modified Syrian hamsters *(Mesocricetus auratus)*, ferrets *(Mustela putorius furo)*, non-human primates (Cynomolgus macaques and Rhesus Macaques) and other mammalian species can be infected to study the pathobiology of COVID-19 (79, 82–86).

Molecular diagnostic test for SARS-CoV-2 were rapidly developed (Table 3). The first Reverse Transcription-Polymerase Chain Reaction (RT-PCR) assay was released by the WHO and targeted three regions of the SARS-CoV-2 genome: the N gene, the E gene, and a highly conserved gene for RNA-dependent RNA polymerase (RdRp) (91, 92). Meanwhile, multiple alternative RT-PCR primer sets are available, while additional methods such as SARS-CoV-2 nucleoprotein antigen tests and antibody detection kits have also been developed (90, 93–95). The SARS-CoV-2 specific antibodies can be detected by rapid diagnostic tests (90). CRISPR-Cas12 based assays, such as DETECTR, identify the presence of SARS-CoV-2 RNA (89, 95). Reverse Transcription Loop-Mediated Isothermal Amplification (RT-LAMP) is a faster (30–40 min) and cheaper alternative for RT-PCR with the advantage of point-of-risk testing (87, 88). Another method is termed Specific High-sensitivity Enzymatic Reporter Unlocking (SHERLOCK) and utilizes Cas13a for the accurate and highly sensitive detection of viral RNA copies (96).

**Table 3:** Detection methods for SARS-CoV-2.

**Type**	**RT-PCR**	**RT-LAMP**	**CRISPR-Cas12**	**Enzyme linked immuno- assay**	**Rapid diagnostic test**
Detection	N gene, E gene, RdRp	N gene, S gene, ORF1ab,	N gene, E gene	IgM/IgG antibodies	IgM/IgG antibodies
Sample type	Nasopharyngeal swab, Oropharyngeal swab	Nasopharyngeal swab, Oropharyngeal swab	Nasopharyngeal swab, Oropharyngeal swab	Plasma or Serum	Plasma or Serum
Time point	Symptom onset	Symptom onset	Symptom onset	Days/weeks after symptom onset	Days/weeks after symptom onset
Advantages	High accuracy, High reliability, Direct detection	High accuracy, High reliability, Rapid detection, Color visualized by the naked eye	High accuracy High reliability	High specificity	Low cost, Ease of use, High specificity
Disadvantages	Labor intensive, Errors with sample collection	Carry-over contamination	High limit of detection	Lower sensitivity	Lower sensitivity
References	(74, 78)	(87, 88)	(89)	(78–80)	(78, 80, 90)

*RT-PCR testing is highly sensitive and widely applied. Limitations are false positive results and prolonged test positivity after recovery from active COVID-19. The characteristics of serology tests for antibodies and innovative CRISPR-Cas methods are shown*.

*RdRp: RNA dependent RNA polymerase*.

## Drugs and Vaccines

### Drugs

#### Chloroquine

Chloroquine is a malaria drug, which passively diffuses into acidic lysosomes, endosomes, and Golgi vesicles. While initial reports and studies were promising for chloroquine/hydroxychloroquine in COVID-19 patients, these findings were not confirmed and the NIH discontinued clinical trials investigating the efficiency of chloroquine (3, 97). In 2020, the FDA had authorized the administration of chloroquine to certain COVID-19 patients, but shortly thereafter, the agency terminated its use due to the high ambiguity regarding its efficiency and side effects (Table 4). In fact, hydroxychloroquine did not improve 28 day mortality in hospitalized COVID-19 patients (1,561 patients, 27% non-survivors) as compared to standard care (3,155 patients, 25% non-survivors) (98). A meta-analysis of studies on the efficiency of chloroquine for treating COVID-19 has shown that there was no significant difference in patient outcome and that the side effects posed a larger threat (99, 107).

**Table 4:** Efforts for drug repurposing.

**Name**	**Chloroquine**	**Azithromycin**	**Remdesivir**	**Dexamethasone**	**Tocilizumab**
Target	Heme polymerase	Ribosomes	RNA-dependent polymerase	Glucocorticoid receptor	Interleukin-6 receptor
Manufacturers	10	18	6	15	2
Efficacy for COVID-19	No	No	Modest or None	Yes	Yes
Side effects	Nausea, Retinopathy, Cardiotoxicity, QT prolongation	Diarrhea, Allergies, Headaches, Liver toxicity	Nausea, Liver toxicity, Anaphylaxis	Gastrointestinal ulcers, Hyperglycemia, Osteoporosis	Headaches, ↑Lipids, Upper Respiratory Infections
References	(82, 98, 99)	(99, 100)	(98, 101, 102)	(103, 104)	(105, 106)

*The medications listed are all FDA approved for other indications and were evaluated in clinical trials for efficacy in severe COVID-19*.

#### Azithromycin

Azithromycin is classified as a broad-spectrum macrolide antibiotic. Azithromycin also amplifies antiviral immune recognition and interferon pathways in airway epithelial cells (108). A single-center study had suggested that a combination of hydroxychloroquine and azithromycin significantly reduced viral loads and time to a negative PCR test after SARS-CoV-2 infection (100). However, subsequent trials failed to reproduce these results, with no significant difference in viral burden as well as continuing positive PCR results (109). The latter study suggests that the antiviral properties of both medications have been overestimated and their side effect profiles might adversly manifest in COVID-19.

#### Remdesivir

Remdesivir was originally designed to target hepatitis C virus, and later studied for effectiveness against Ebola virus. It is classified as an anti-viral adenosine-tri-phosphate analog, which is incorporated into the forming viral RNA chain by the RNA-dependent polymerase and disrupts viral replication (98). Remdesivir showed some efficacy in inhibiting infection of mammalian cells by human coronaviruses (110). In clinical trials, remdesivir tended to shorten the recovery time for adult patients and reduced symptoms of upper respiratory infection (101).

Remdesivir was one of the first drugs granted emergency use authorization, and it is now approved by the FDA for use in adults children (>12 years) for the treatment of COVID-19 requiring hospitalization (102). However, remdesivir only achieves modest benefits for subgroups of hospitalized COVID-19 patients.

#### Dexamethasone

Dexamethasone is a potent anti-inflammatory corticosteroid that binds to the glucocorticoid receptor and depending on the dosage either reduces the expression of certain pro-inflammatory genes or boosts the transcription of a subset of anti-inflammatory regulators (103). A meta-analysis of *n* = 1,703 severely ill COVID-19 patients found glucocorticoids to reduce 28 day mortality (32% vs. 40%) without an increased risk for severe adverse events (111). The RECOVERY trial (*n* = 2,104) showed that dexamethasone decreased COVID-19 mortality (29% vs. 41%) in patients on mechanical ventilation or receiving oxygen without mechanical ventilation (23 vs. 26%) (104). No difference in survival was found in patients who did not require respiratory support. Hence, dexamethasone is recommended for severe cases of SARS-CoV-2 infection and its best role could be as part of a combination therapy (22).

#### Tocilizumab

Tocilizumab is a humanized monoclonal antibody against the Interleukin-6 (IL-6) receptor used in autoimmune diseases and inflammatory disorders (112). Clinical trials suggest that Tocilizumab can reduce hyperinflammation during severe COVID-19. More specifically, one trial showed that Tocilizumab reduced mortality of COVID-19 when compared to standard care while increasing the risk of secondary infections (105). Tocilizumab relieves clinical symptoms, reduces the requirement for supplementary oxygen, reverses lymphopenia and decreases C-Reactive Protein (CRP) levels (106). A direct positive correlation was found between CRP levels, lung lesions and higher severity of COVID-19 (113). While not all studies have shown significant differences in disease severity or survival of infected patients treated with Tocilizumab compared to a placebo (114), a meta-analysis of *n* = 2,120 patients supported a reduction of mortality in severe cases of COVID-19 (115). In a more recent analysis of *n* = 4,116 adults, Tocilizumab reduced COVID-19-associated mortality (29% vs. 33%) and was more effective in combination with glucocorticoids (54% vs. 47%) (116). Patients receiving Tocilizumab were less likely to require mechanical ventilation and showed improved clinical outcomes (116). Sarilumab is another blocking anti-IL-6R antibody which is studied for COVID-19.

#### Immunoglobulin

Neutralizing antibodies and passive immunization are a feasible approach to mitigate SARS-CoV-2 infection (117, 118). Passive immunization could be especially helpful for immunocompromised individuals at risk for severe clinical manifestations such as respiratory failure (119). Prophylaxis against infectious agents using purified polyclonal immunoglobulin (Ig), also known as polyvalent immunoglobulin, is not a new idea (120). Ranging from highly specific to very broad, neutralizing monoclonal antibodies have been designed against a variety of viral agents such as MERS-CoV (121, 122).

Recent work on the antibody repertoire produced by infected humanized mice and recovered patients has generated a large bank of antibodies that can be used against COVID-19. Anti-SARS-CoV-2 spike antibodies were generated by immunizing mice with a DNA plasmid encoding the RBD protein. In addition, B-cells were isolated from the peripheral blood of recovered patients (117).

The antibodies generated from both studies were reported to be highly similar in function and efficacy against many spike variants. However, four of them, utilized individually or in cocktails, showed promising results against newer strains that had originated from human populations (117). A cocktail therapy was proposed to limit viral resistance to therapy by using antibodies that target two distinct, non-overlapping regions of the RBD (123). Nevertheless, the antibodies were not effective in neutralizing SARS-CoV-2 when new spike mutations arose from *in vitro* passaging or when combinations of antibodies that target overlapping regions were administered (123). Other neutralizing antibodies (LY-CoV555 and LY-CoV016) have shown promising results in the BLAZE-2 clinical trial. Bamlanivimab (LY-CoV555) alone reduced the risk of symptomatic COVID-19 by 80% (124), while in a separate study the combination of Bamlanivimab (LY-CoV555) with Etesevimab (LY-CoV016) was found to decrease hospitalization and death from COVID-19 by 70% (124–127). Furthermore, Regeneron's REGN-COV2 neutralizing antibody cocktail (Casirivimab and Imdevimab) was effective in reducing the viral load in patients with delayed immune responses or with high initial virus titers (128).

### Vaccines

**Figure 3:**
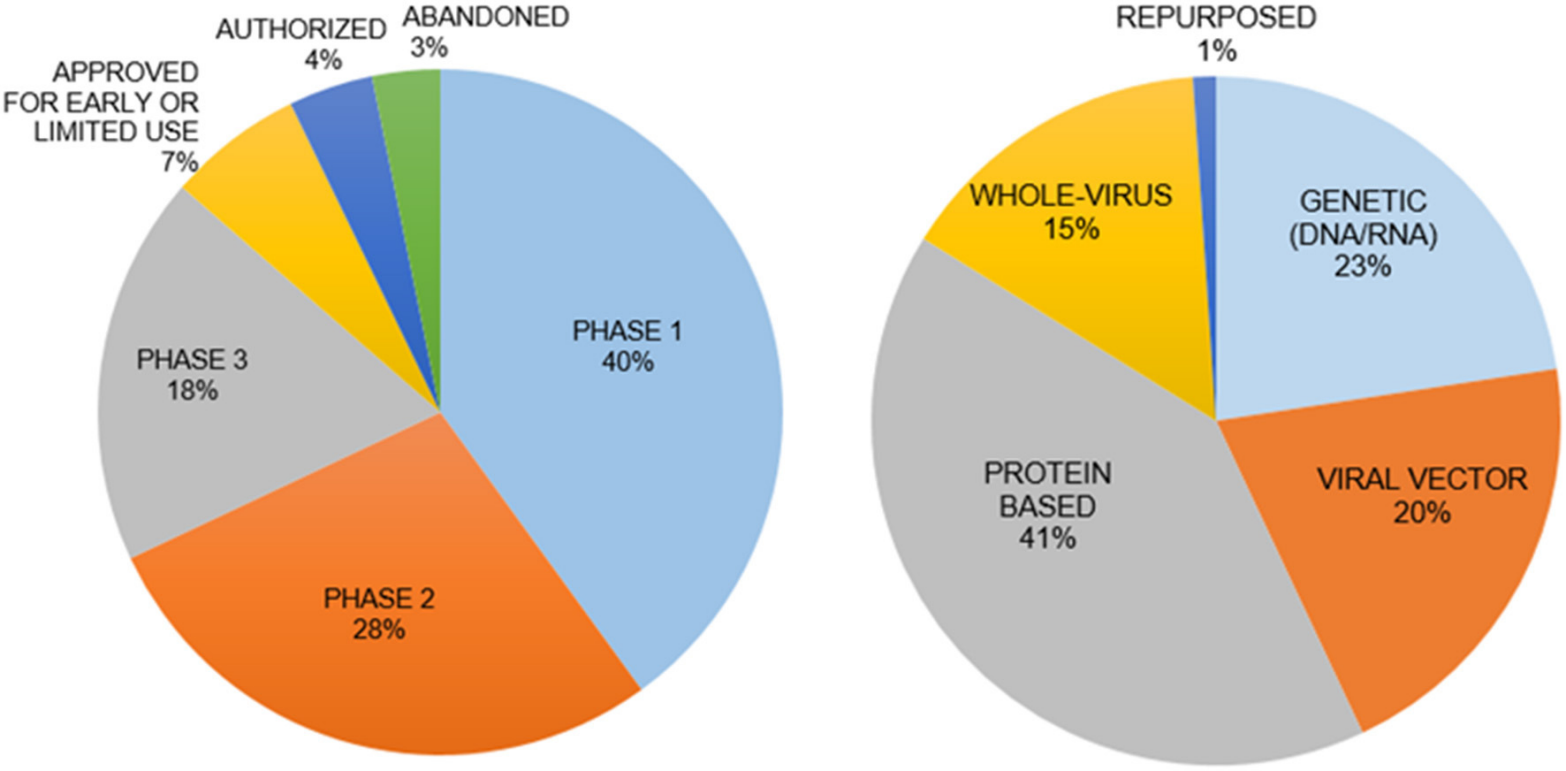
Vaccine types and clinical phases of their development. The genetic vaccines immunize with mRNA for SARS-CoV-2 and protein-based vaccines immunize with spike protein to induce immunity. The interim analysis of phase 3 clinical trials for both types of vaccines have been disclosed with promising results in November 2020. The mRNA vaccines are a new principle with little information on how long the induced immunity will last.

Vaccines are the best approach for prevention of infection. There are five types of vaccines under development (Figure 3) (129):

(i) Genetic Vaccines use SARS-CoV-2 specific DNA/RNA sequences to stimulate an immune response. (ii) Viral Vector Vaccines employ alternative viruses as “carriers” for SARS-CoV-2 genes. (iii) Whole Virus Vaccines present an inactivated form of the virus to the immune system. (iv) Protein-based Vaccines incorporate selected virus proteins such as the spike protein. (v) Repurposing the Bacillus Calmette-Guérin vaccine to stimulate the immune system. Recent discussions raised questions regarding the safety, long-term side effects, and social implications of a vaccine developed in a short period time without sufficient pre-clinical testing and adequate clinical trials. A major concern for a vaccine is the syndrome of acquired cellular immunopathology, a condition observed when the delivery platform for the viral proteins or genes leads to a violent pro-inflammatory response from T-cells. This results in the migration of white blood cells into target tissues, further deteriorating the health of a patient. Another concern is Antibody-Dependent Enhancement (ADE), when non-specific antibodies generated by the vaccine allow for enhanced viral internalization, thus potentially worsening the infection and pathophysiology of COVID-19 (130). A vaccine could also have low efficacy in terms of long-lasting protection from infection because of insufficient neutralizing antibodies, weak memory T cell responses or new SARS-CoV-2 variants.

In the pre-COVID-19 era, vaccine development lasted on average about a decade and required extensive funding, scientific diversity, and countless volunteers. The federal Center for Biologics Evaluation and Research is a branch of the FDA responsible for evaluating the safety and efficacy of novel medication and vaccines. The FDA and CDC have established a strict set of clinical trials (phase 1–3), through which the safety and efficacy are investigated. In the last year, there has been a race to develop the first vaccine to prevent COVID-19.

There are more than 110 potential vaccine candidates, with over 80 in human trials and almost another 80 vaccine candidates in preclinical testing. There are currently seven approved vaccines, developed mainly in the US, Russia, China, India, UK, Germany and Belgium. In some cases, the development of vaccine candidates came to a halt, such as for Merck, Imperial College London, Themis, Institut Pasteur and IAVI (131–136) (Figure 3).

The clinical trial of the mRNA-based vaccine (BNT162b2) from Pfizer/BioNTech enrolling 43,000 participants showed a reduced risk for SARS-CoV-2 infection by over 90%. This vaccine received FDA emergency approval in the US, while it has been fully approved in other countries (137). Further studies have shown that the BNT162b2 vaccine has 95% efficacy in preventing a COVID-19 infection 7 days after the second dose (137, 138). Of note, the nanoparticles that deliver the mRNA contain polyethylene glycol (PEG), a compound that has been linked to unwanted severe allergy-like symptoms. Similar concerns have been raised with the nanoparticles used in the mRNA vaccine from Moderna. It consists of mRNA-1273 encapsulated in lipid nanoparticles (139). The mRNA-1273 showed similar efficiency (94%) to the Pfizer vaccine and was granted FDA emergency use authorization. The protection by a protein-based adenovirus vector vaccine from AstraZeneca was around 70% with some uncertainties about optimal dosing and recent concerns about a risk for thrombotic complications. Regardless of these promising results, the duration of long-lasting immunity induced by these vaccines has yet to be determined. The Johnson & Johnson vaccine also uses an adenovirus vector to express SARS-CoV-2 spike protein in the host cells to induce immunity. This process yields SARS-CoV-2-specific antibodies in ~90% of individuals after the first dose (140). The Johnson & Johnson vaccine has been associate with a very rare risk for cerebral venous sinus thrombosis. Mild to moderate local (e.g., pain and swelling at injection site) and systemic (e.g., fever, chills) side effects are very common for the current COVID-19 vaccines. Seropositive participants develop higher antibody titers and experience higher rates of systemic side effects (141). It is expected that SARS-CoV-2 will eventually transition from a pandemic to an endemic disease, a change that is associated with the distribution of infected individuals. Endemic dynamics are characterized by a shift of primary infections to younger ages in the population, which for COVID-19 usually causes only mild disease or asymptomatic infection. The shift to mild endemic disease depends on the rate of virus transmission and may be accelerated by vaccination (142).

## Nanomedicine Approaches

Nanomedicine approaches may provide new solutions in the fight against COVID-19. The hope is that nanotechnology can improve the effectiveness and specificity of drugs and vaccines. The nanomedical field utilizes nanomaterials: particles in the nanometer scale that possess unique chemo-physiological properties. Two key characteristics of nanoparticles are their size and polarity. Their size, ranging from 10 to 100 nm, allows them to easily interact with a biological target of similar size and pass through several types of membranes, such as the lung-blood vessel junction and the blood-brain barrier (143). In addition, the polarity of nanoparticles can be modified to facilitate a specific purpose such as binding other drugs, increasing the surface stability, or reducing aggregation and precipitation (144, 145). Specialized nanoparticles with a magnetic nature can be guided through the body via a system of external magnets and forced to increase their temperature by exposing them to an oscillating magnetic field, a technique currently used in oncology for tumor suppression (146–148). Moreover, these particles can be both organic and inorganic, used individually or aggregated, and combined with other medication or other nanoparticles. Due to the unique features of nanomaterials, widespread applications in both the prevention and treatment of SARS-CoV-2 are feasible. Nanotechnology could be applied for personal protective equipment, gene silencing, creating biosensors, developing pharmacologically active compounds and nano-vaccines, and for directly destroying SARS-CoV-2 particles (149–151).

Biosynthesis of nanoparticles by microorganisms has recently emerged as an alternative to conventional chemical and physical synthesis. Biosynthetic nanoparticles can have similar morphology and properties to their conventional counterparts (152, 153). There are several benefits of large-scale synthesis of microbe-derived nanoparticles such as avoiding hazardous chemicals, expensive reagents or toxic materials for stabilization and synthesis. Nanoparticles can bioconjugate, genetically engineer, infuse, mineralize or even assist in self-assembly of viral and bacterial particles. These techniques could be used as tools for vaccine design and production (152, 153).

There are several designs for nanoparticle-based peptide vaccines. Nanoparticles can be used to construct a multiple antigen-presenting platform. Self-assembling lipo-peptides, consisting of a lipid chain bound to an antigen, can form micelles with enhanced epitope presentation ability (154). Another safe and effective method of antigen delivery to antigen-presenting cells is encapsulation or conjugation of antigens with nanoparticles in order to preserve their structure and protect them from degradation (155). Nanoparticles designed to either deliver antigens or act as adjuvants can be administered intranasally to induce immunity against lower respiratory tract virus infections, such as influenza, RSV and adenovirus (155). Bacteriophage-derived nanoparticles from Escherichia virus Q-beta were incorporated into a H1N1 vaccine of high immunogenicity and low safety concerns in a phase 1 clinical trial (156).

Adenovirus (class I-dsDNA virus), adeno-associated virus (AAV, class II-ssDNA virus), human papilloma virus (HPV) or even human immunodeficiency virus (HIV) can be modified into carriers for targeted gene/protein delivery (153, 157–161). Bacteria can be engineered for nanoparticle biosynthesis such as *Bacillus cereus* and *Bacillus subtilis* for silver nanoparticles (162), *Pseudomonas aeruginosa* and *Pseudomonas fluorescens* for gold nanoparticles (163), *Shewanella algae* for platinum nanoparticles (164), and *Pseudomonas aeruginosa* for Lanthanum nanoparticles (165).

### Nanoparticle Applications

Nanotechnology is a fast growing industry. The current $60 billion market is expected to double to $120 billion in 5 years. The main market prospects involve the utilization of nanoparticles for medicine, food, agriculture, conductors and computers. In medicine, nanoparticles are used and developed for applications inside of the body (e.g., drug delivery, repair of tissues) and for external purposes.

**Figure 4:**
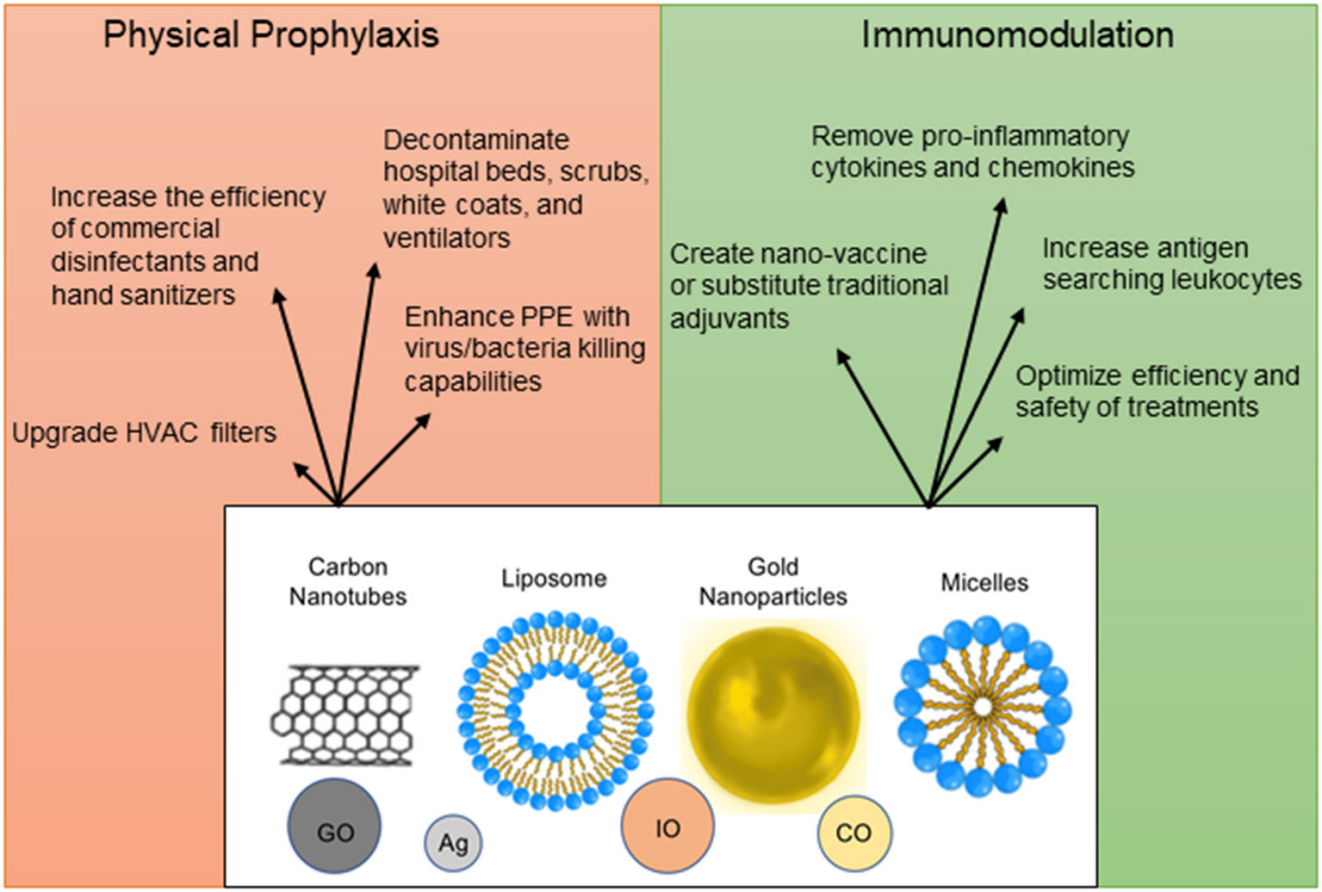
Nanoparticle applications as prophylactic and therapeutic measures. Nanoparticles can be used in a plethora of ways for protection against infection, for immunomodulation, vaccine design, and optimization of detection methods. The structural design of nanomaterials is diverse and includes engineering of carbon nanotubes [Graphene Oxide (GO)], liposomes, micelles, precious metals [Silver (Ag), Copper Oxide (CO), and Iron Oxide (IO)].

To decrease the spread of SARS-CoV-2, nanoparticles with a potential to inactivate the virus can enhance physical barriers, sterilize commonly contacted surfaces or air filters, and be incorporated into hand sanitizers and disinfectants (166–169) (Figure 4). Personal Protective Equipment (PPE) such as masks and gloves could be upgraded with nanoparticles that have antimicrobial or antiviral capabilities. Iron-oxide nanoparticles (IO-NPs) and Silver nanoparticles (Ag-NPs) have been shown to neutralize various strains of Influenza and Coronaviruses by physically binding to the SARS-CoV-2 virion and preventing internalization into host cells (166, 167, 170, 171). Moreover, Copper Oxide nanoparticles (CO-NPs) possess antimicrobial capabilities against a plethora of respiratory tract pathogens such as *Staphylococcus aureus* and *Pseudomonas aeruginosa* (172). Antimicrobial nanoparticles use chemical and biological mechanisms to eliminate microbes such as cell membrane disruption, DNA and protein damage, gene silencing, heavy metal ion toxicity, Reactive Oxygen Species (ROS) formation, and prevention of biofilm formation (173).

One of the strongest arguments for the use of nanoparticles in drug enhancement is that modern drug delivery can lack target specificity due to a poor cellular uptake, insufficient stability under physiological conditions, non-target effects, and excessive immunogenicity (174). A novel approach to avoid such problems uses short interactive RNA molecules bound to nanoparticles, which can interact with a biomarker on the desired cell population, thus localizing the drug's effects to avoid unnecessary contact with other cells and reduce overall toxicity (175, 176). Nanoparticles coated with specific antibodies against a cellular receptor such as human ACE2 or against SARS-CoV-2 spike protein comprise an elegant delivery system for any drug that requires cell specificity and may help reduce the dose of medication and off-target side effects (174). Many nanoparticle types can be used, such as polymers, dendrimers and quantum dots. Nanobots, microscopic robots that can carry out localized drug delivery, could be controlled by a user and might advance drug delivery even further in future (176).

The properties of the molecules mentioned above could also be engineered to reduce the chances of secondary infections that are associated with COVID-19 pathophysiology. PPE, patient gowns, scrubs, white coats and commonly contacted surfaces could be coated with a mixture of nanoparticles to protect healthy and infected individuals. Cotton fabrics can be enriched with zinc oxide nanoflowers to trap and denature SARS-CoV-2 spike protein (177). Additionally, enhancing conventional hand sanitizers and upgrading air filter systems with antimicrobial nanoparticles could be useful for disinfection and containment of SARS-CoV-2 spread. FDA-approved iron-oxide nanoparticles (IO-NPs) were recently found to bind to the envelope and spike protein subunits of SARS-CoV-2 and alter their conformation, thus inactivating the virus (178). Nanoformulations can help to reduce the needed quantities of precious elements such as gold, silver and copper.

The SpyCatcher/SpyTag technology allows irreversible conjugation of a recombinant protein by adding a sequence of the SpyTag peptide (13 amino acids) to its DNA sequence. The SpyTag spontaneously reacts with the SpyCatcher protein and allows for oligomerization (179). This system was employed to generate mosaic nanoparticles that display multivalent antigens of SARS-CoV-2 spike RBD along with RBDs from different animal betacoronaviruses to enhance B cell responses and elicit high-titers of cross-reactive, neutralizing antibodies (180).

### Nanoparticle Applications for Detection, Immune Prophylaxis, and Vaccines

#### Nanoparticles in Diagnostics

Early and rapid detection is key for lowering the basic reproduction number of infected individuals. Nanoparticles can be engineered as biosensors for the detection of biomarkers, including nucleic acids (DNA, RNA), specific antigens (proteins, enzymes), or antibodies in order to rapidly and accurately detect SARS-CoV-2 (143, 149, 181, 182). Recent advancements in nanotechnology have allowed for the release of a SARS-CoV-2 detection platform that uses graphene conjugated to an anti-spike antibody. This novel kit requires no sample pretreatment or labeling and is impressively effective in detecting SARS-CoV-2 at very low concentrations (183). Alternative detection methods have been designed such as dual-functioning plasmonic biosensors, which tap into the energetics of DNA-RNA hybridization, as well as Graphene Oxide particles coated with fluorophore-bound DNA target strands that can detect viral helicase (184, 185).

#### Nanoparticles for Drug Delivery and Vaccines

Nanoparticles can be engineered to directly target SARS-CoV-2 or as immunomodulatory factors to prime and alarm the immune system and reduce the inflammatory response during COVID-19.

Small-interfering RNAs against conserved regions of SARS-CoV-2 were incorporated into lipid nanoparticle formulations and upon delivery into lungs suppressed viral replication and improved survival of infected mice (186).

Graphene Oxide Nanoparticles (GO-NPs) have been shown to increase leukocyte numbers such as macrophages and T cells. This effect boosts adaptive immunity, thus allowing for a better immune response and viral clearance, or a possible use as vaccine adjuvants. In the scenario of uncontrolled hyperinflammation, nanodiamonds elicit an anti-inflammatory state in macrophages, while carbon and graphene sheets can be repurposed to remove pro-inflammatory cytokines and interleukins from the blood of patients (149).

Most importantly, nanotechnology may offer solutions to some of the major problems of traditional vaccines and medications such as sensitivity to acidity, water insolubility, or absorption. Nanoparticles can increase drug delivery efficiency by binding or encapsulating hydrophobic or pH-sensitive drugs and creating a targeted release. For example, certain nanoparticles bound to drugs can be modified using organic molecules that provide better release characteristics, such as Cholesterol-modified-Hydroxychloroquine. Other nanoparticles can facilitate the transport of two or more drugs, thus decreasing each dose as well as the side effects, while augmenting the combined outcome (187).

Another proposal claims that a simple and unconventional vaccine design could combine layered double hydroxide (LDH-NPs) nanoparticles and a plasmid encoding short hairpin RNA to silence the expression of targeted genes, such as essential SARS-CoV-2 proteins to stop infection early. The LDH-nanoparticles are compatible with mammalian cell lines and can insulate the shRNA against degradation, thus providing a promising delivery mechanism (188).

The current COVID-19 mRNA vaccines (Moderna, Pfizer/BioNtech) contain mRNA wrapped in lipid nanoparticles. More nanoparticle vaccines are under development. For example, NVX-CoV2373 (Novavax) is a recombinant nanoparticle-based vaccine, which incorporates the full trimeric spike glycoprotein with a saponin-based adjuvant (Matrix-M1) (189). Testing on macaques and later in phase 1–2 human clinical trials revealed that this vaccine could elicit neutralizing antibodies such as anti-spike IgG antibodies, as well as a specific T-cell response (189).

Sinovac Biotec Company also designed a nanovaccine against SARS-CoV-2 and successfully tested it in mice. This NP based vaccine incorporates the RBD subunit of the spike protein combined with two adjuvants: Monophosphoryl Lipid A (MPLA) and CpG-ODN, which stimulate TLR4 and TLR9, respectively. The vaccination of mice was achieved in three stages (original shot and two boosters) and resulted in a potent and protective T cell response accompanied by neutralizing IgA antibodies (190). This nanovaccine is currently in clinical phase 3 testing and in light of the promising results, a large-scale manufacturing plant is under construction (191).

Of note, another nanotechnology vaccine was recently found to induce a persistent antibody production and long-lasting memory response for at least 7 months in mice (192). In this vaccine design, the RBD of spike protein was conjugated via the SpyTag/SpyCatcher technology to ferritin nanoparticles. Hence, the unique capabilities of nanoparticles could revolutionize the processes of vaccine design, manufacture, and delivery.

### Challenges and Limitations

While the widespread use of nanoparticles in medicine is an exciting idea, a few drawbacks may delay the realization of these endeavors. An overall examination of literature surrounding the design and application of nanoparticles in pharmacology has shown that there is a lot of variability between the results of independent research studies, and translating the efficacy of these particles from an *in vitro* to an *in vivo* situation is difficult (2). Additionally, critics have emphasized that the large-scale production of nanoparticles will be a high hurdle to overcome, especially when trying to keep these treatments affordable (193). The required sophistication of the manufacturing processes of nanoparticles and intellectual property rights can drive up their prices, although overall health care expenditures could be saved if nanomaterials and nano-vaccines accomplish to prevent COVID-19. Another limitation of nanoparticles are risks of unwanted tissue interactions and toxicity, unwanted spread and deposition in the body including unwanted crossing of the blood-brain barrier (194, 195). Accidental inhalation into the lungs is feared to cause epithelial injury, pulmonary inflammation and contribute to fibrosis depending on the size and chemical composition of the nanoparticles (196). Moreover, nanoparticles have been shown to interfere with biological processes like inflammation, oxidative stress, mitochondrial function, macrophage phagocytosis and platelet function (2). Acute or chronic toxicity of nanoparticles may be caused via ROS generation, cell membrane binding, DNA damage, altered cell cycle regulation and protein denaturation (197). Another important issue is the incomplete understanding of long-term effects of nanoparticles in humans and the environment. For example, a study on the effect of chronic administration of nanoparticles to rats resulted in structural damage in their testis, including disorganization of spermatogenic cells, misoriented testis and reduction of germ cells (198, 199). Allergic reactions and anaphylaxis to the mRNA lipid nanoparticle vaccines (Moderna, Pfizer/BioNtech) for COVID-19 have been blamed on the nanoparticle design and composition (200).

These limitations, and other unknown risks, should be taken into consideration when evaluating the actual potential of nanoparticles to form a reasonable approach toward nanomedicine.

## Conclusions

Nanotechnology is an emerging field that can alter the way we approach the diagnosis, treatment, and prevention of human diseases. Nanomedicine offers unique potentials to address future epidemiological challenges with other emerging viruses. The ongoing COVID-19 pandemic has shown that health care systems were underprepared for such a large-scale event. Nanotechnology seems very promising, but one must not forget that it is a young and unexplored field. The current state of the field leans in favor of nanoparticles supporting modern medicine, but risks and long-term side effects remain hard to assess.

Effective therapies of COVID-19 remain elusive, but fortunately, the widespread public distribution of vaccines has begun. The promising potential of nanoparticles is not limited to diagnostic and therapeutic approaches but can also be applied to global prophylactic measures that aim toward limiting the spread and symptoms of SARS-CoV-2 infection. Theranostics, a new discipline of medical science, focuses on detecting and eliminating new viral or bacterial threats using nanomedicine and nanodrugs for diagnostics and therapy. This field has demonstrated futuristic applications of nanotechnology, such as spike protein-specific nanoparticles and neutralizing nanomaterials. It may even become a pharmacological standard of care once the side effects are well-understood and mitigated. While true benefit of nanomedicine in the fight against COVID-19 remains to be seen, it is worthy of in-depth considerations and efforts.

## Author Contributions

AS and KK wrote the manuscript and prepared the figures. MB wrote and edited the manuscript and supervised and funded the work. All the authors are responsible for the contents of this publication.

## Conflict of Interest

The authors declare that the research was conducted in the absence of any commercial or financial relationships that could be construed as a potential conflict of interest.
